# Maple Quality Testing: An Evaluation of Maple Syrup Compliance with Grade A Standards

**DOI:** 10.3390/foods14244216

**Published:** 2025-12-08

**Authors:** Emmanuel Owoicho Abah, Mark Isselhardt, Mark Cannella

**Affiliations:** 1 Department of Extension, University of Vermont, Burlington, VT 05405, USA; eabah@uvm.edu (E.O.A.); mark.isselhardt@uvm.edu (M.I.); 2 The Food Systems Research Institute (FSRI), University of Vermont, Burlington, VT 05405, USA

**Keywords:** maple syrup, grade A, quality standard, golden grade, dark grade, grade compliance, color, flavor

## Abstract

This study assesses the quality of pure maple syrup sold online to consumers. It aims to determine how likely consumers are to receive syrup that meets legal grading standards. Grade A clarity, color, density, and flavor tests were performed using visual color comparison to known standards, refractometry, and sensory analysis, respectively. The lowest compliance rates were observed in the color test for Golden syrup samples, with 1.7% in 2021, 26.4% in 2023, and 21.9% in 2024. The 2021 test was delayed due to COVID-19, which increased cases of color darkening. Flavor compliance was the second lowest, with Dark samples exhibiting compliance rates of 79.6% in 2021, 79.5% in 2023, and 73.9% in 2024, due to their ability to mask flavor, making it difficult for producers to detect. Syrup color often fails Grade A standards, especially for Golden syrup, likely due to producer grading issues and post-packaging darkening. The producer’s ability to accurately evaluate flavor differs between Golden and Dark syrup, with higher observed flavor compliance for Golden syrup and lower compliance due to off-flavors in Dark syrup, as observed in this study. Using proper instruments to verify defects, accuracy, and limitations is essential, and consistent staff training and unbiased feedback are vital in preventing human error.

## 1. Introduction

Maple syrup is a natural sweetener derived from boiling and concentrating the sap of maple trees, primarily *Acer saccharum* (sugar maple) and *Acer rubrum* (red maple) [1,2]. Its composition consists mainly of sucrose (about 96%), with trace amounts of hexoses and minerals. Maple syrup has evolved from a local sweetener to a globally recognized product with significant economic impact [3,4,5,6]. In 2022, Canada’s maple syrup industry provided 12,582 full-time jobs, contributed C$1.13 billion to the GDP, and generated C$235 million in taxes [7]. The United States’ maple syrup crop value generally ranges from $150 million to $210 million annually. It remains a crucial economic sector in the Northeastern states, supporting rural jobs and income through direct, indirect, and induced contributions [8,9,10,11,12]. Canada remains the leading producer, accounting for roughly 73% of global output, followed by the United States, with Vermont, New York, and Maine as the primary producers [13]. Key importers include the United States, Germany, the United Kingdom, Japan, and Australia, reflecting the product’s growing international reach and branding as a natural, sustainable sweetener [13].

Quality standards for maple syrup generally align with the principles defined by the United States Department of Agriculture (USDA) and the Canadian Food Inspection Agency (CFIA). The USDA standards for Grade A pure maple syrup is a voluntary classification system that requires syrup to have a density between 66.0 and 68.9 °Brix, a characteristic maple flavor free of off-flavors, uniform color, and clarity [14]. Many U.S. States have their own regulations for pure maple syrup. Some states, like Vermont, have regulations which are mandatory for syrup sold in the retail marketplace. Additionally, Vermont and New Hampshire have adopted a higher minimum density of 66.9 °Brix [15]. This density standard applies to all syrup sold directly to consumers in Vermont regardless ofwhere it was produced.

Syrup is classified into four color grades: Golden Delicate > 75.0% light transmittance (Tc), Amber Rich 50.0–74.9%Tc, Dark Robust 25.0–49.9%Tc, and Very Dark Strong < 25.0% Tc, based on light transmittance at 560 nm [2,14,16,17]. These grading parameters are crucial for consumer perception, trade, and regulatory compliance. The sensory and physicochemical quality of maple syrup can deteriorate through several mechanisms. Pre-processing and processing factors such as microbial growth in raw sap, prolonged storage before boiling, variations in processing temperature, and pH influence the development of color and flavor [18,19,20,21]. Excessive heating or oxidation during packaging and storage can cause the syrup to darken. Contamination at any stage of sap harvest or processing can lead to the formation of undesirable flavors [22]. Inadequate filtration or poor equipment sanitation can also result in turbidity, sediment formation (“sugar sand”), or microbial contamination [23,24]. Maintaining the correct density is essential as u syrup below the density standard may ferment, while syrup above the density standard can lead to crystallization and color degradation [25,26,27].

Compliance with quality standards is a continuing challenge for this high-value specialty sweetener. Recent reports from North American regulatory agencies have identified inconsistencies in maple syrup grading, particularly concerning color and flavor [28,29]. In Canada, out-of-compliance syrup inventories rose from 11% in 2010 to 21% in 2018, reflecting the growing difficulty of maintaining quality during high-volume production and variable environmental conditions [30]. Given the globalization of the syrup trade and the rise in online marketing, assessing product compliance is increasingly relevant to both consumer protection and market integrity. Despite extensive research on maple syrup processing and composition [31,32,33], there remains a limited understanding of how retail maple syrup, especially products sold online, aligns with official grading standards. This study, therefore, assesses the compliance of consumer-purchased pure maple syrup with established quality standards, focusing on color, clarity, density, and flavor. The primary research questions are:

1. Which type of compliance infractions are most or least common in the marketplace?
2. How likely is a consumer to receive an out-of-compliance maple syrup product from an online purchase?
3. Are compliance levels observed at the same or different rates between two different color grades of syrup within the same year?
4. Are compliance trends similar between different years using the same online product search terms?
5. What quality standards, education, and best practices can improve syrup quality in the marketplace?
6. The study hypothesis is that the overall color compliance level in a specific year will differ between two syrup color grades. Secondly, any density compliance infractions are more likely to occur at lower densities than at higher densities. The null hypothesis is that no difference is detected.

## 2. Materials and Methods

### 2.1. Sample Purchase and Preparation

**Figure 1:**
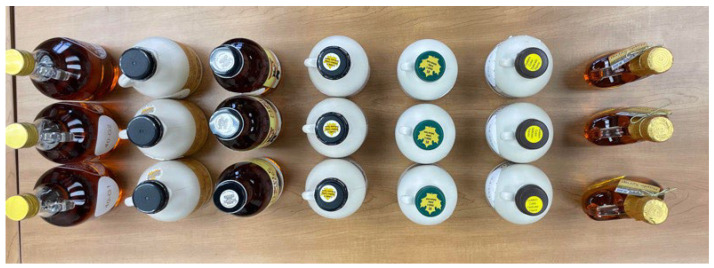
Maple syrup container sizes and labels observed from online sample purchases.

This study tested two commercial grades of pure maple syrup: Golden-Delicate and Dark-Robust. Retail products were collected over three sampling years (2021, 2023, and 2024). A total of 507 syrup samples were obtained from approximately 30 independent U.S. businesses (by year) through purchases from online retail vendors. Sampling years and sample counts were: 245 in 2021, 182 in 2023, and 80 in 2024. To ensure product and producer diversity, syrups were sourced from businesses located primarily in New England and Northeastern states. Each sample was packaged in pint-sized containers as commercially sold with exceptions for glass packages measured in similar metric units (Figure 1). Observational data on product labeling (graded/ungraded), packaging type, batch codes, and shipping conditions were recorded. Samples were stored under controlled conditions before analysis. In 2021, due to COVID-19–related delays, half of the samples were refrigerated between 33–36 °F, and the remainder were kept at room temperature (64–68 °F) due to limited facility capacity. Analytical procedures followed consistent protocols across all study years to ensure comparability of syrup quality and grade determinations.

### 2.2. Clarity Standard Instrumentation

**Figure 2:**
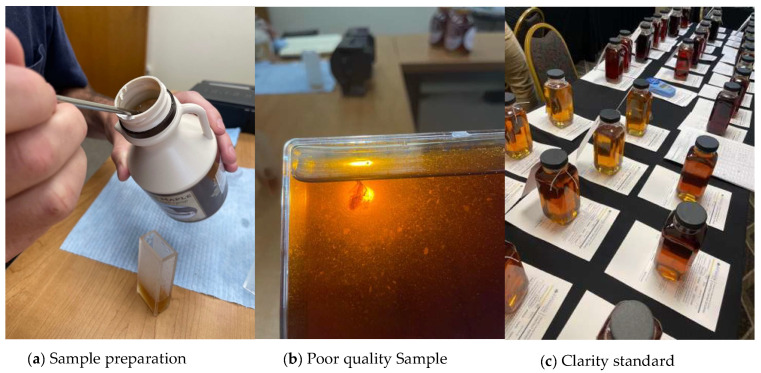
Pictorial presentation of maple syrup clarity test preparation (**a**), poor quality sample (**b**), and clarity standard (**c**).

Clarity is assessed by visually inspecting syrup samples in transparent bottles. The standard requires packaged syrup to be “clean, clear, and free of any dirt”. Syrup was poured into a clear plastic container and held towards either an overhead fluorescent light or an outdoor window for visual inspection. The visual representation of the clarity examination process is presented in Figure 2.

### 2.3. Color Standard Measurement

**Figure 3:**
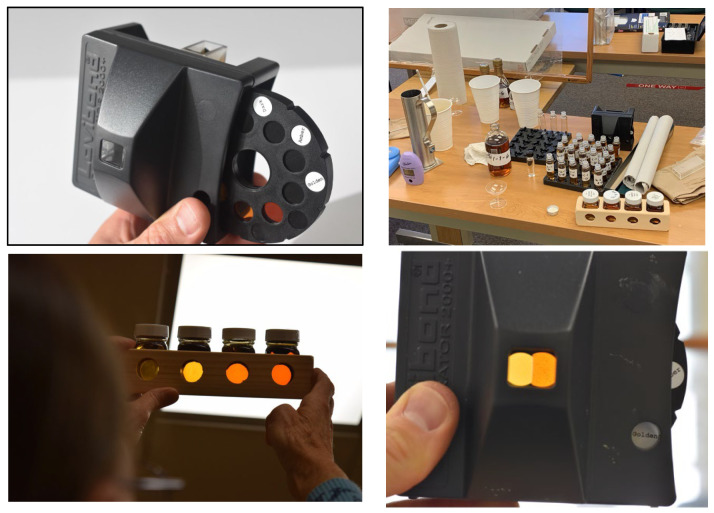
Available color test procedures include the Lovibond color comparator (**top left** and **bottom right**), the Hannah Digital Grader (**top right**), and a temporary color kit (**bottom left**).

Maple syrup color was evaluated according to USDA grading standards, which classify syrup into four color grades based on percent light transmittance (LT) at 560 nm using a spectrophotometer (or an equivalent method) [14]. In 2021, syrup color was evaluated using three methods: temporary color kits, a Hanna Checker digital grader (Model HI 759), and a Lovibond color comparator. This multi-instrument approach allowed cross-validation of grading consistency. From 2023 onward, the Lovibond comparator was used exclusively, as it employs permanent glass standards and provides greater accuracy than the digital grader. The device uses an amplifying prism system that overlaps the sample and comparison fields for accurate color matching, with a 40 mm path-length sample cell. The prism optics are ultrasonically sealed to prevent contamination, and measurements are taken under a constant, standardized light source. Color classification was achieved by rotating the reference disk until the closest match between the syrup sample and color standard was obtained (see Figure 3).

### 2.4. Density Standard Measurement

**Figure 4:**
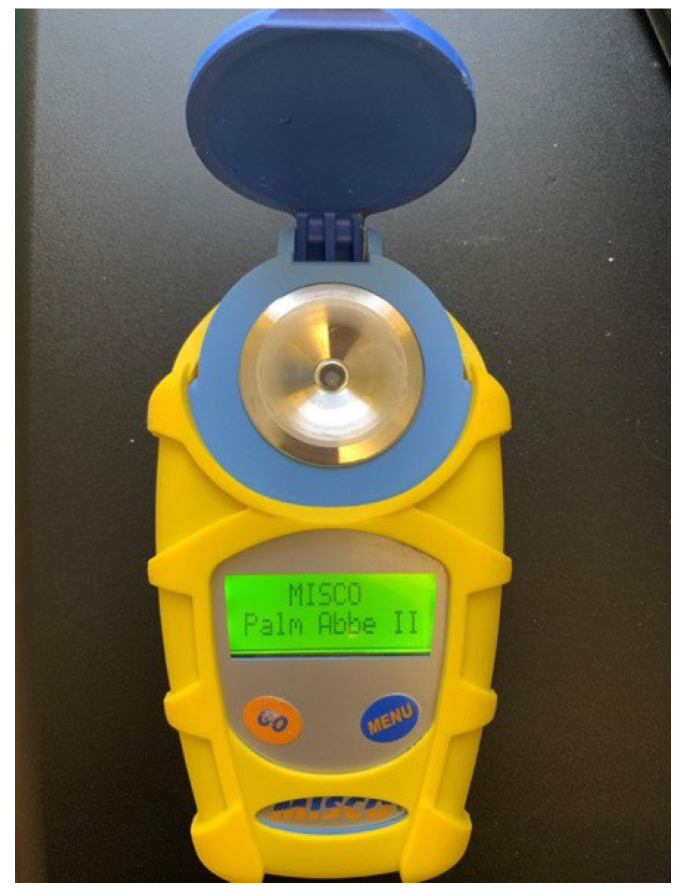
A MISCO (Palm Abbe II) digital refractometer was used to test syrup density.

In 2021, the density of each sample was measured using a hydrometer with temperature correction and a digital refractometer. Pure maple syrup must have a density of 66.9–68.9 °Brix to comply with Vermont regulations (minimum 66.0 °Brix USDA regulations). The MISCO digital Brix refractometer was used in all three years of this study (Figure 4). In this study, the acceptable margin of error was set at ±0.2 °Brix.

### 2.5. Flavor Standard Measurement

A panel of maple syrup specialists from the University of Vermont (UVM) and an agency representative trained in grading pure maple syrup evaluated the aroma and flavor of each sample. UVM maple specialists have completed training in the “Flavor Profile Method of Sensory Analysis” and are qualified to serve as professional descriptive panelists. Before testing, panelists went through a standardized calibration process to ensure consistent sensory performance across all study years. Training included a review of the USDA (2015) Grade A flavor definitions [14], a joint tasting session using verified reference standards representing common maple off-flavor descriptors such as “metabolism”, “buddy,” “fermented,” “scorched,” and reference standards for acceptable flavor in the grades tested for this study. Panelists practiced identifying defects and intensities using the same sample sets. To ensure consistency across years, annual recalibration sessions were held before each new testing cycle. The same panelists were used for the three years studied. Panel performance was tracked to ensure agreement through discussion-based consensus checks and comparison to established reference standards. All flavor assessments adhered to the USDA (2015) Grade A compliance procedure for syrup flavor evaluation [14], and responses were documented using standardized terminology consistent with production guidelines.

### 2.6. Statistical Analysis

Statistical analyses were performed using RStudio software version 2025.05.0+496. Descriptive statistics summarized the distribution of categorical variables. Associations between grade, flavor category, defects, and compliance outcomes were assessed with Chi-square or Fisher’s Exact Tests. The Chi-square test was used when expected cell counts met its assumptions, while Fisher’s Exact Test was applied for comparisons involving small or unevenly distributed categories, such as low-frequency defects or unbalanced year-to-year samples. Using both tests ensured valid inference across a dataset with variable sample sizes and sparse contingency tables. Statistical significance was set at *p* < 0.05.

## 3. Results

### 3.1. Maple Syrup Origin

**Figure 5:**
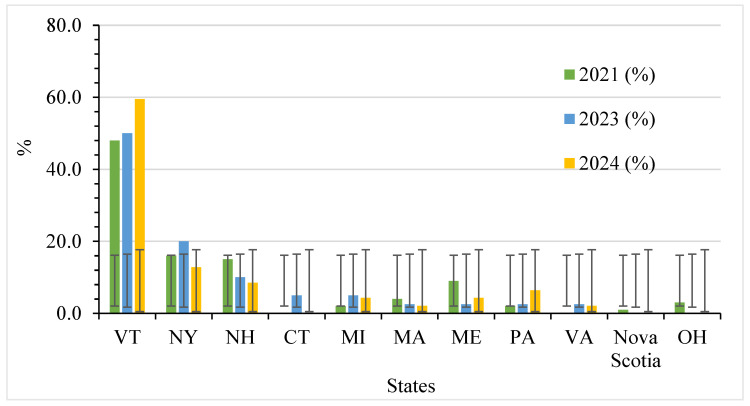
Distribution of the maple syrup sample origin with standard deviation bars. Note: Golden (Delicate): *n* = 116 (2021), 91 (2023), 32 (2024); Dark (Robust): *n* = 129 (2021), 91 (2023), 48 (2024). Note: VT = Vermont, NY = New York, NH = New Hampshire, CT = Connecticut, MI = Michigan, MA = Massachusetts, ME = Maine, PA = Pennsylvania, VA = Virginia, OH = Ohio.

Maple syrups were obtained from different sellers in ten U.S. states and one Canadian province. Figure 5 shows purchased maple syrup samples from several states and one province.

### 3.2. Clarity Standard Analysis

**Figure 6:**
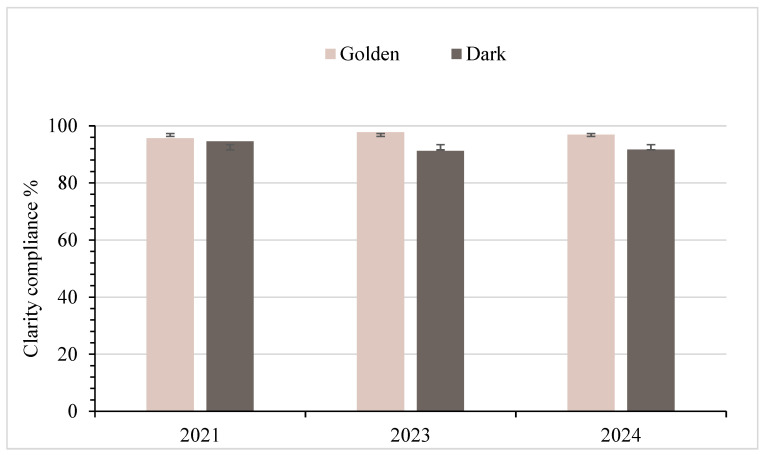
Percentages of syrup in compliance with grade A standards for clarity, with standard deviation bars. Note: Golden (Delicate): *n* = 116 (2021), 91 (2023), 32 (2024); Dark (Robust): *n* = 129 (2021), 91 (2023), 48 (2024).

The clarity compliance rate consistently remained high over the three years for Golden samples (95.7% in 2021, 97.8% in 2023, and 96.9% in 2024), averaging 96.8% compared to the Dark samples, which averaged 92.5% (Figure 6). The maple syrup clarity analysis explored whether clarity outcomes differed across years (2021, 2023, and 2024) for Golden and Dark syrups using Fisher’s Exact Test because of low cell counts. The test showed *p*-values of 0.789 for Golden syrup and 0.566 for Dark syrup, both above the 0.05 threshold, indicating no significant difference in sampling year or clarity between the two syrup types. When assessed within each year (Golden vs. Dark), none of the *p*-values met the 0.05 threshold (0.914, 0.1, and 0.643, respectively, for 2021, 2023, and 2024), as shown in Table 1.

### 3.3. Color Standard Analysis

**Figure 7:**
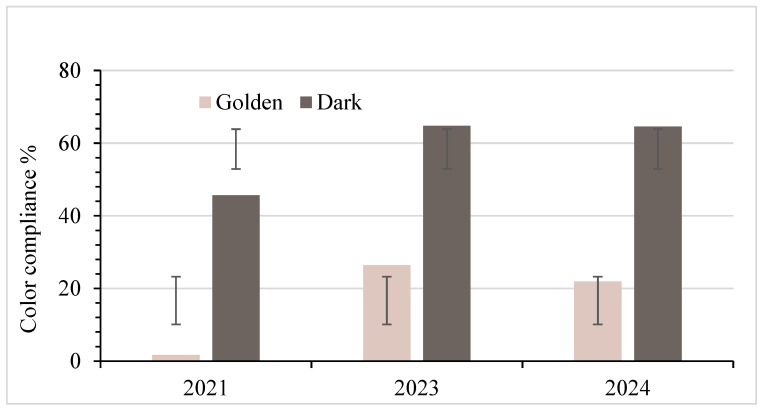
Percentages of syrup in compliance with grade A standards for color, with standard deviation bars. Note: Golden (Delicate): *n* = 116 (2021), 91 (2023), 32 (2024); Dark (Robust): *n* = 129 (2021), 91 (2023), 48 (2024).

The color test analysis for 2021 was significantly delayed due to interruptions in laboratory access during the COVID-19 lockdown. This delay partly contributed to the darkening of the syrup, thereby influencing the color test results that year. Figure 7 showed that 1.7%, 26.4%, and 21.9% of the samples from 2021, 2023, and 2024, respectively, met the Grade A color standard for Golden. Despite study limitations in 2021, the prevalence of color non-compliance in 2023 and 2024 demonstrates a significant challenge for the industry in ensuring that the labeled color grade complies with the regulation. This could necessitate additional operating costs for reprocessing the large volumes of samples that do not meet grade A standard (processing grade).

Compared to the Golden samples, the color compliance of the Dark grade samples is higher, at 45.7%, 64.8%, and 64.6% in 2021, 2023, and 2024, respectively. To test the hypothesis, the Chi-square test was used for Golden syrup, and Fisher’s exact test was applied to Dark syrup due to sample size differences. At the 5% significance level, an analysis comparing syrup color between the Golden and Dark categories revealed significant differences for each year: 2021 (*p* = 8.94 × 10^−18^), 2023 (*p* = 4.19 × 10^−7^), and 2024 (*p* = 4.33 × 10^−4^), as shown in Table 1. Additionally, when assessing each color category across the three years, notable variations were observed for both Golden (*p* = 6.57 × 10^−8^) and Dark syrups (*p* = 7.46 × 10^−3^). This indicates that syrup color compliance differed consistently between Golden and Dark grades in every year and that each grade also showed meaningful changes in compliance across the three-year period.

### 3.4. Density Standard Analysis

**Figure 8:**
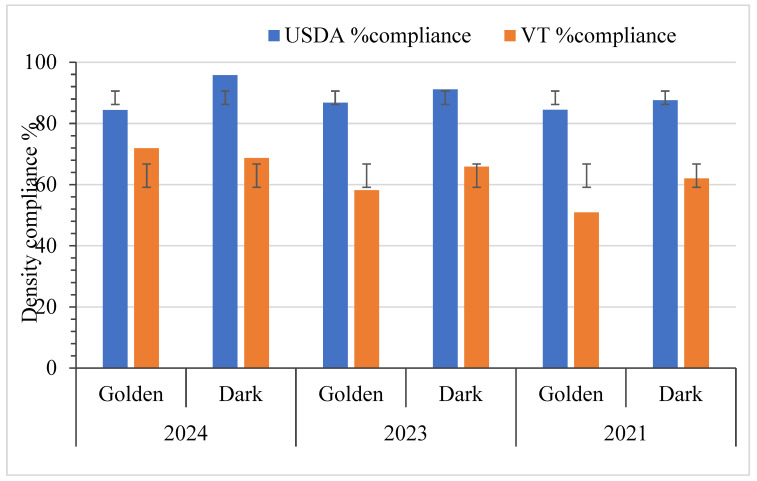
Compliance (%) for grade A density (USDA and Vermont standards) between Golden delicate and Dark maple syrup samples (2021–2024) with standard deviation bars. Note: Golden (Delicate): *n* = 116 (2021), 91 (2023), 32 (2024); Dark (Robust): *n* = 129 (2021), 91 (2023), 48 (2024).

**Figure 9:**
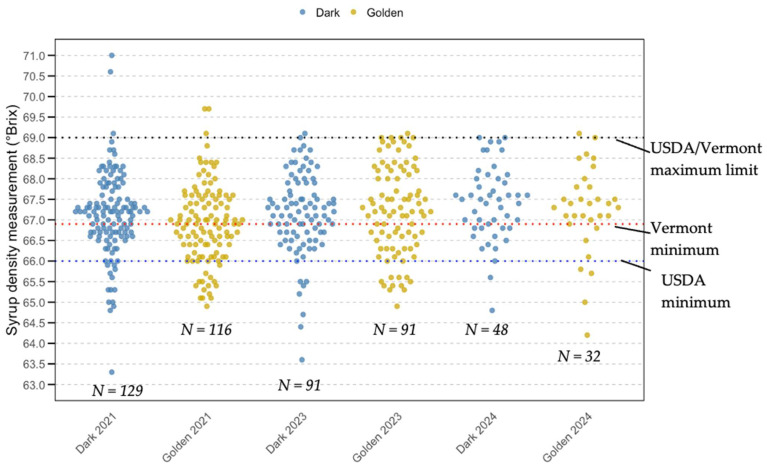
Comparison of syrup density between Golden delicate and Dark maple syrup samples (2021–2024). Note: Golden (Delicate): *n* = 116 (2021), 91 (2023), 32 (2024); Dark (Robust): *n* = 129 (2021), 91 (2023), 48 (2024).

Figure 8 and Figure 9 show sample densities (Dark and Golden grades) measured in °Brix and compliance percentages for both the USDA Grade A density standards and the Vermont density standard. It highlights the differences in compliance rates between the USDA and Vermont standards. As shown in Figure 8, in 2021, 84.5% of Golden syrup met USDA density standards, and 50.9% met Vermont density standards (VT). While Dark syrup achieved 87.6% (USDA) and 62% (VT). By 2023, Golden syrup improved to 86.8% (USDA) and 58.2% (VT), while Dark syrup is 91.2% (USDA) and 65.9% (VT). In 2024, Golden syrup recorded 84.4% (USDA) and 71.9% (VT), compared to Dark syrup’s 95.8% (USDA) and 68.7% (VT). Overall, Dark syrup consistently outperformed Golden syrup in meeting density standards. As illustrated in Figure 9, Dark syrup densities ranged from 63.3 to 71.0, while Golden syrup densities ranged from 64.9 °Brix to 69.7 °Brix. Statistical comparisons using contingency tables and Fisher’s or Chi-squared tests showed no significant differences in syrup density distribution. At the 5% significance level, there were no significant differences in density observed between Golden and Dark syrup in any of the individual years: 2021 (*p* = 0.604), 2023 (*p* = 0.477), and 2024 (*p* = 0.109), as shown in Table 1. Additionally, density remained statistically stable across the three years for each color category, with *p*-values of 0.875 for Golden syrup and 0.271 for Dark syrup. Figure 8 demonstrates that instances of low-density compliance are primarily due to syrup density falling below the minimum acceptable level, thus the study hypothesis on density is accepted.

### 3.5. Flavor Standard Analysis for Grade A Samples

**Figure 10:**
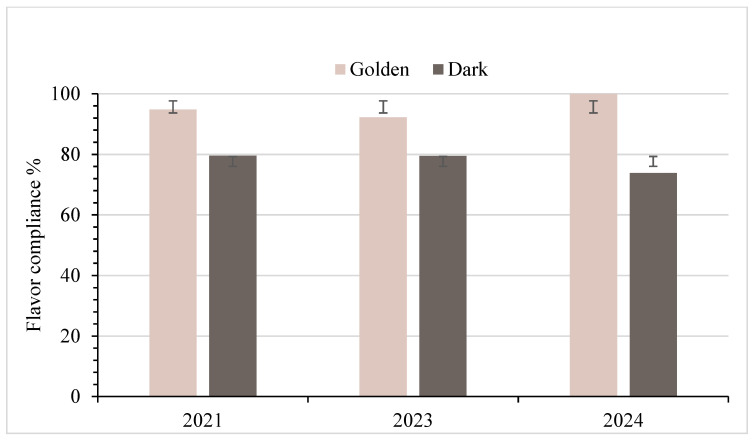
Percentages of syrup in compliance with grade A standards for flavor, with standard deviation bars. Note: Golden (Delicate): *n* = 116 (2021), 91 (2023), 32 (2024); Dark (Robust): *n* = 129 (2021), 91 (2023), 48 (2024).

Figure 10 shows that Golden samples maintained high flavor compliance in each year evaluated, with rates of 94.8% in 2021, 92.2% in 2023, and 100% in 2024. The consistently high compliance may reflect the more delicate flavor profile of Golden syrup, which makes defects easier to detect. For Dark samples, flavor compliance was moderately high in each year, measured at 79.6% in 2021, 79.5% in 2023, and 73.9% in 2024. As indicated in Table 1, statistical tests revealed significant flavor differences between Golden and Dark syrups at the 5% level in each year tested: 2021 (*p* = 0.003), 2023 (*p* = 0.015), and 2024 (*p* = 0.001), indicating consistent distinctions by color grade. Within each grade, no significant differences were detected among years, with *p*-values of 0.246 for Golden syrup and 0.557 for Dark syrup. These findings indicate that flavor characteristics differ between grades, while remaining stable within a given grade over time.

### 3.6. Degree of Non-Compliance and Effects of Packaging Material

**Figure 11:**
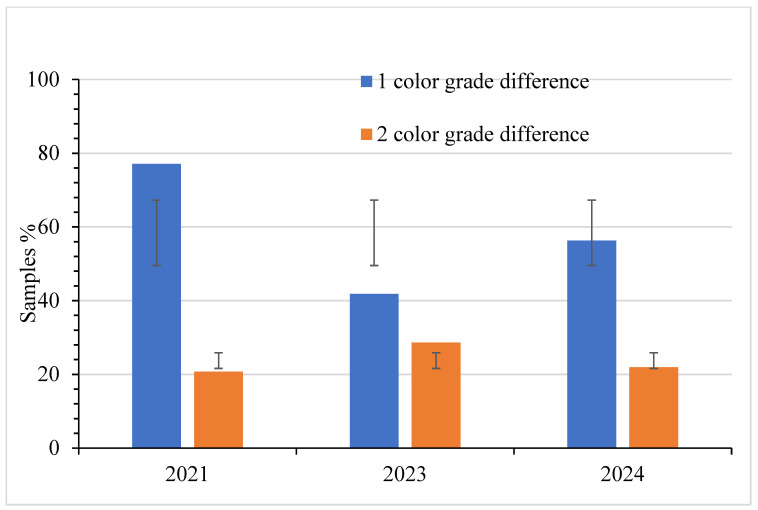
Degree of color difference after observing the syrup samples sold as Golden delicate syrup, with standard deviation bars. Note: Golden (Delicate): *n* = 116 (2021), 91 (2023), 32 (2024); Dark (Robust): *n* = 129 (2021), 91 (2023), 48 (2024).

Figure 11 illustrates the extent of color non-compliance. Cases involving a 1-grade difference were the most common, accounting for 77.6% of cases in 2021 and 56.3% in 2024. In contrast, cases of a 2-grade difference appear moderate over the three years (20.7% in 2021, 28.6% in 2023, and 21.9% in 2024). Nevertheless, these cases are significant because they highlight the extent to which the syrup darkens after packaging. Golden samples that failed to meet color and VT density standards made up 48.3% in 2021 and 25.0% in 2024. For Dark samples, the percentages were 19.4% in 2021 and 12.5% in 2024. The use of plastic packaging containers was predominant. Dark syrup samples packaged in plastic containers accounted for 93.3% in 2021 and 45.1% in 2024, respectively. For Golden syrup samples, these figures were 87.0% in 2021 and 52.9% in 2024, respectively.

## 4. Discussion

### 4.1. Clarity Standards

The high level of compliance with clarity standards observed in the tests shows that clarity, a highly valued characteristic, was almost entirely maintained by producers, especially with Golden samples. This compliance demonstrates strong, stable quality, with only minor fluctuations. The light color of the Golden samples makes problems easier to detect visually than the Dark samples. This indicates that concerns remain about the detectability of clarity problems in Dark and Very Dark syrup samples. The dark color of the Dark grade can hinder producers from detecting cloudiness. In contrast, the Golden grade’s light appearance (high light transmittance) likely facilitated the identification and removal of cloudiness before packaging, thereby reducing the number of out-of-compliance products sold online. Daily processing variations, such as filter conditions and rinse water volume, can also affect syrup clarity [22]. 

Filtration is the critical step in maple syrup production that removes sediments and sugar sand while maintaining syrup quality and clarity. Producers have relatively few filtration options, typically using cloth and paper filters for smaller operations, or filter presses with diatomaceous earth for larger producers. Producers achieve a relatively high rate of compliance with clarity expectations, indicating the robustness of existing practices. The statistical analysis of clarity data from 2021, 2023, and 2024 showed no significant differences between Golden and Dark syrup in any year (*p* = 0.914, 0.100, and 0.643, respectively). Although the 2023 comparison approached significance (*p* = 0.100), it did not meet the 0.05 threshold. Similarly, when evaluated across years, neither Golden (*p* = 0.789) nor Dark syrup (*p* = 0.566) exhibited statistically significant variation in clarity. The application of Fisher’s Exact Test to Golden syrup, given low cell counts, reinforces the need for cautious interpretation in some cases. Nonetheless, these results affirm that clarity, a key visual quality parameter, remains stable across syrup grades and production years, reflecting consistent production practices and effective quality control.

### 4.2. Color Standards

Statistical analysis revealed significant differences in color compliance between Golden and Dark maple syrup within each testing year. Golden syrup consistently showed the lowest compliance rates, with the most pronounced deviations occurring in 2021. These early results should be interpreted cautiously, as COVID-19-related laboratory access restrictions caused prolonged delays between sample acquisition and testing. During this period, samples were stored under mixed conditions, room temperature and refrigeration, which likely accelerated oxidative darkening, especially in HDPE plastic containers with higher oxygen permeability. This explains much of the reduced color-compliance rate observed in Figure 7.

The mechanisms underlying such darkening are well documented. Morselli and Whalen (1989) noted that syrup stored in bulk or retail containers can darken due to (1) blending syrups with differing invert sugar contents, (2) oxygen-driven oxidation within partially permeable containers, and (3) the inherent instability of reverse-osmosis–concentrated sap [34]. These processes, combined with extended storage during the pandemic, likely contributed to the elevated darkening observed in 2021. The economic implications of storage-related darkening are substantial, as a shift in hue that no longer fits the assigned grade can diminish market value and consumer trust [34]. Bosley et al. (2020) [35] reported that syrup stored in uncoated plastic jugs darkened at approximately 2.6% LT per month, whereas syrup stored in coated jugs darkened at approximately 0.8% LT per month. Reheating syrup prior to packing also lowers LT through oxidation reactions [35]. Unfortunately, coated containers are no longer available to producers. The significant overall *p*-value across years (6.57 × 10^−8^) further confirms that grade-based differences are systematic rather than random.

In 2023, Golden and Dark syrups showed a significant difference in color compliance (*p* = 4.19 × 10^−7^), reflecting considerable variation in producers’ ability to achieve the correct color grade across categories. Just like in 2021, Dark syrup had higher compliance than Golden syrup, indicating that Golden syrup was more prone to deviations in light-transmittance standards. This pattern persisted in 2024, with a continued significant difference in compliance between Golden and Dark syrups (*p* = 4.33 × 10^−4^). Although overall compliance improved from 2021, Golden syrup still lagged behind Dark syrup in meeting USDA color standards, confirming that Golden syrup remains the more challenging grade for producers to produce and market within the required specifications over the years. Therefore, due to these statistically significant differences in color compliance, consumers buying Golden syrup are much more likely to receive a product that does not meet the USDA color standard than those purchasing Dark syrup.

The observed pattern of color non-compliance, particularly the darkening of Golden syrup, is consistent with earlier studies showing that syrup darkens during long-term storage [36] or when stored in oxygen-permeable packaging [34]. Sendak (1982) [37] demonstrated that HDPE allows far greater oxygen and CO_2_ transmission than glass, driving color development and pH change. However, CO_2_ permeability is higher, and atmospheric oxygen is the dominant cause of oxidative darkening [37]. Field studies such as those reported by Walters and Yawney (1978) showed darker, more variable syrup from sap collected through improperly installed plastic tubing [38], and Perkins et al. (2020) showed that uncoated containers darkened syrup more than three times faster than coated ones [35]. Long-term storage trials similarly found grade loss and flavor decline in warm conditions and significantly greater darkening in plastic than in glass or metal, primarily when temperature interacted with container type [39,40].

Although improvements in sap handling have reduced microbial-related darkening, Morselli and Whalen (1991) found that aseptic tapping consistently produced lighter syrup [21]. The persistent non-compliance of Golden syrup in this study suggests that current issues arise primarily from post-production handling, packaging (oxidation), or grading practices rather than sap collection. The 2024 results confirm that Golden syrup again had significantly higher color non-compliance than Dark syrup (*p* = 4.33 × 10^−4^). These findings underscore the need for improved quality assurance in grading accuracy, packaging selection, and storage management, particularly for the lightest syrup grade.

### 4.3. Density Standards

Ensuring that the density remains within an acceptable range is crucial for compliance with Grade A standards and for avoiding any quality issues arising from density that is too low (fermentation) or too high (crystallization). The density tests indicated that most results falling out of compliance were at the lower end of the density spectrum, specifically at 66.0 °Brix and 66.9 °Brix for the USDA and Vermont standards, respectively. Syrup below the lower density spectrum can support microbial growth and associated food safety risks, as well as result in a viscosity that appears “watery” to consumers. Henderson (2009) reported that Canadian producers packaged syrup below the legal density, suggesting that syrup with a density below 66 °Brix poses legal, quality, economic, and safety risks, including fermentation or molding, which can lead to additional reprocessing costs, unsellable products, customer loss, and recalls [41]. Isselhardt and Cannella (2024) [15] reported that significant compliance issues, such as failing to achieve a 66.0 °Brix concentration, may arise from inaccurate syrup temperature measurements during production or packaging. In a situation when syrup temperature is lower than the producer believes it to be, the hydrometer will float at a higher point and appear to the producer to be denser than it truly is, which would result in syrup being removed from the boiling evaporator prematurely [15].

Statistical analysis showed no significant differences in density compliance between Golden and Dark syrup in 2021 or 2024. Within each grade, year-to-year variations were also not significant (Golden: *p* = 0.875; Dark: *p* = 0.271), suggesting stable density control practices across time and syrup types. This consistency reflects the practical application of standardized boiling and evaporation methods. These results also suggest that producers are not intentionally attempting to sell more syrup as Golden Delicate even if it does not meet the color grade. Bulk syrup buyers pay a higher price for Golden Delicate syrup compared to the darker grades, but such a price premium is not consistently observed for retail syrup sales. However, marginally lower compliance observed in specific Golden syrup batches, for example, 18 out of 116 in 2021, warrants ongoing monitoring due to the stricter consumer expectations and sensory sensitivity associated with this grade. In 2024, no statistically significant difference was found between Golden and Dark syrups in density compliance, indicating that syrup grade does not meaningfully affect the likelihood of consumers receiving an in- or out-of-compliance product. Of the samples determined to be out of grade for density, it was far more likely that they were below the standard than above it. This suggests an issue with producers accurately measuring syrup temperature, leading to syrup cooling enough to affect hydrometer readings.

### 4.4. Flavor Standards

Flavor evaluation showed that Golden syrup met compliance standards at very high rates, with some samples achieving complete compliance. Dark syrup, by contrast, displayed lower compliance, suggesting persistent challenges in identifying and managing flavor defects in this grade. The difference may reflect the greater ability of darker syrup to mask off-flavors, or an issue of producer education as to the sensory qualities of acceptable Dark syrup versus Dark syrup with a distinct defect. The flavor quality of maple syrup is influenced by factors such as tree species, sap chemistry, collection timing, and processing methods. The flavor of maple syrup is also affected during evaporation through complex reactions involving sugars, amino acids, minerals, and other precursors. Clean processing equipment and strict sanitation remain critical, and small residues in sap lines or tanks can alter the flavor profile during boiling. However, defining what constitutes a “good” maple flavor is challenging because no formal standards exist, and the wording of syrup quality standards lacks specificity. Producers often rely on personal experience to judge the acceptability of flavor, and their exposure to syrup from other operations varies widely. This variability likely influences the consistency and accuracy of flavor grading, particularly for more complex syrups produced late in the season.

Fisher’s exact test was used to assess flavor compliance due to the small sample sizes in 2024. Statistically significant differences were observed between Golden and Dark syrups in each year (2021: *p* = 0.003; 2023: *p* = 0.015; 2024: *p* = 0.001), with Dark syrup showing consistently lower compliance rates. For example, in 2021, 21% of Dark samples had flavors that failed to meet the minimum Grade A flavor standards, compared to only 7% of Golden samples. These findings suggest a strong correlation between syrup grade and flavor quality. However, within-grade comparisons across years showed no significant difference (Golden: *p* = 0.246; Dark: *p* = 0.557), indicating stability in flavor compliance within each syrup type despite seasonal variability. This suggests that consumers are likely to consistently receive appropriate, compliant flavors when purchasing Golden syrup products, but may experience more off-flavors when purchasing Dark-Robust syrup. Nevertheless, the persistently lower performance of Dark syrup highlights opportunities for improvement, particularly in handling, boiling, and filtration practices during late-season production. The most frequent market infraction observed was low compliance with color standards, often due to darkening after packaging, but flavor remains a critical quality factor for consumer satisfaction. To improve overall syrup quality, producers would benefit from targeted education on grading standards, quality control tools, and best practices in filtration, evaporation, and storage. Intake inspection and consumer awareness can further reinforce adherence across the supply chain. The significant difference in 2024 flavor compliance between Golden and Dark syrup (*p* = 0.001) supports the hypothesis that syrup grade impacts a consumer’s likelihood of receiving a compliant product. The null hypothesis is therefore rejected. This result may also reflect the complexity of assessing Dark syrup, as certain flavor variations can be more challenging to distinguish from off-flavors. Providing additional training and resources could help producers develop greater consistency in evaluating what is and is not acceptable flavor within the Dark grade.

### 4.5. Best Practices for Maple Compliance Testing

Managing environmental and procedural factors during production and packaging is crucial for reducing maple syrup darkening. Limiting excessive headspace in containers helps minimize oxidation and preserve the syrup’s color. Assigning a darker grade as you approach a color threshold can help prevent color compliance issues caused by slight color variations. These measures help producers meet standards while maintaining visual appeal and quality.

Producers should adjust density-testing targets to prevent the sale of low-density syrup and ensure they accurately measure the syrup temperature at which the hydrometer floats. Although packaging syrup at the lowest allowable density may yield the highest possible financial return, a slightly higher target will allow a reasonable margin for error. While this adjustment could reduce revenue, it might be acceptable at specific volumes. Therefore, finishing the syrup above 66.9 °Brix (for sales to Vermont) ensures it meets consumer and regulatory expectations. For accurate syrup density measurements, producers should use validated hydrometers and keep multiple instruments on hand to ensure reliability. Dirty, damaged, or cold hydrometers can lead to errors, and regular monitoring helps identify problems early. However, it is essential to acknowledge that most hydrometers used for maple production are designed with a precision of only 1.0 °Brix. This inherent limitation means readings should be interpreted with caution and, where possible, supplemented with higher-precision instruments or cross-checks. Careful attention to these details helps prevent over- or under-concentration, both of which can affect flavor, spoilage, crystallization, and overall usability.

Clarity is crucial for producing high-quality maple syrup, but no quantitative measure or tool exists to determine whether a sample meets the standard. To ensure clarity, select an appropriate filtration method and strictly follow the manufacturer’s guidelines, as this process removes sediments that can detract from the syrup’s clarity and consistency.

For flavor, the most critical step in accurate grading is experience in tasting a wide range of syrups. Given that most maple producers do not purchase syrup from others, the potential for tasting many different examples of syrup is low. Assembling a team of specialists with broad tasting experience can help achieve accurate and comprehensive sensory evaluations, which would be ideal but not necessarily practical for the average maple producer. Tasting at the time of production runs the risk of sensory, flavor, and aroma saturation, which can make a detailed analysis of syrup flavor difficult. Syrup samples can be collected per batch and tested for flavor compliance at a separate location, preferably at a different time. Both high-quality and off-flavor reference samples can assist in flavor grading.

Collaborating with other producers in grading provides valuable insights into industry standards and helps refine internal processes. Incorporating batch codes will help minimize losses due to quality issues, as they are easily identifiable. Using appropriate instruments to verify defects, accuracy, and limitations is crucial. Regular staff training and unbiased feedback are indispensable in preventing human error. Creating an inventory directory enables effective monitoring and quality control, allowing producers to identify areas for improvement and efficiently manage recalls when necessary.

## 5. Conclusions

Syrup color remains the most common area of noncompliance with Grade A standards, with Golden Delicate syrup showing disproportionately low color compliance issues, indicating that consumers buying Golden syrup are much more likely to receive a product that does not meet the USDA (and, in Vermont, legally required) color standard compared to those purchasing Dark syrup. These differences are likely due to post-packaging darkening, limitations in producer grading accuracy, and the Golden Delicate’s greater sensitivity to small changes in light transmittance. Flavor assessment also shows grade-dependent patterns: Golden Delicate syrup generally meets expected flavor profiles, while Dark robust syrup is more prone to off-flavors. In contrast, the clarity test showed compliance across grades, reflecting effective filtration practices. Density issues occur less often but seem related to differences between USDA and Vermont minimum thresholds and the limited precision of hydrometers, which may cause some syrups to fall just below the legal standard.

Because maple syrup is a high-value specialty sweetener, the industry recognizes that regulatory compliance and consistent sensory quality are essential to maintaining market reputation. Documenting retail-level compliance issues, including specific deficiencies in color, flavor, clarity, and density, is necessary for producers and sellers to improve production and grading practices and ensure a reliable consumer experience. Improving compliance requires targeted quality-control measures such as batch coding, which allows producers to trace and correct storage-related deviations; routine verification using reliable tools such as the Lovibond comparator for color and standardized sensory reference sets for flavor, to support more consistent grading; and ongoing training, which ensures that producers and staff can accurately identify defects and correctly apply USDA and Vermont grade definitions. It is also essential to cross-check grading sessions with neighboring producers or extension specialists to align individual grading practices with industry norms. Additionally, structured inventory monitoring, tracking container type, storage duration, and environmental conditions, enables timely interventions, including product rotation or recall when necessary.

The study faced several limitations, including inconsistent documentation of clarity-grading criteria. The reliance on subjective sensory evaluation, which required multiple evaluators and constant comparative sampling to resolve borderline flavor cases, is an approach that can lead to sensory fatigue and introduce variability. Statistical limitations also emerged from uneven sample sizes, necessitating the use of both Chi-square and Fisher’s exact tests. Future research should explore sensor-based, computer vision, or machine learning systems to reduce subjectivity in color and flavor assessment, thereby improving precision and supporting regulatory compliance. Strengthening these evaluation tools and grading practices is critical to ensuring that all syrup sold in Vermont, regardless of origin, meets Vermont’s legally enforceable quality standards and delivers a consistent, trustworthy product to consumers.

## Figures and Tables

**Table 1:** Summary of statistical significance (*p*-values at 5% significance (0.05)) for quality attributes of Golden and Dark syrup tested for 2021, 2023, and 2024.

Attribute	Year	Golden vs. Dark	Golden 3 Year Average	Dark 3 Year Average
Clarity	2021	0.914	0.789	0.566
	2023	0.1		
	2024	0.643		
Flavor	2021	0.003	0.246	0.557
	2023	0.015		
	2024	0.001		
Color	2021	8.94 × 10^−18^	6.57 × 10^−8^	7.46 × 10^−3^
	2023	4.19 × 10^−7^		
	2024	4.33 × 10^−4^		
Density	2021	0.604	0.875	0.271
	2023	0.477		
	2024	0.109		

## Data Availability

The original contributions presented in the study are included in the article, further inquiries can be directed to the corresponding author.
